# Mind-stimulating leisure activities: Prospective associations with health, wellbeing, and longevity

**DOI:** 10.3389/fpubh.2023.1117822

**Published:** 2023-02-17

**Authors:** Dorota Weziak-Bialowolska, Piotr Bialowolski, Pier Luigi Sacco

**Affiliations:** ^1^Centre for Evaluation and Analysis of Public Policies, Faculty of Philosophy, Jagiellonian University, Cracow, Poland; ^2^Human Flourishing Program, Institute for Quantitative Social Science, Harvard University, Cambridge, MA, United States; ^3^Department of Economics, Kozminski University, Warsaw, Poland; ^4^Dipartimento di Scienze Filosofiche, Pedagogiche ed Economico-Quantitative, University of Chieti-Pescara, Pescara, Italy; ^5^metaLAB (at) Harvard, Harvard University, Cambridge, MA, United States; ^6^Consiglio Nazionale delle Ricerche - L'Istituto di Scienze del Patrimonio Culturale, Naples, Italy

**Keywords:** reading, word and number games, playing cards, health outcomes, emotional wellbeing, cognitive impairment, SHARE, longevity

## Abstract

**Introduction:**

This study examines prospective associations within a 6-year perspective between three mind-stimulating leisure activities (relaxed and solitary: reading; serious and solitary: doing number and word games; serious and social: playing cards and games) and 21 outcomes in (1) physical health, (2) wellbeing, (3) daily life functioning, (4) cognitive impairment, and (5) longevity domains.

**Methods:**

Data were obtained from 19,821 middle-aged and older adults from 15 countries participating in the Survey of Health, Ageing, and Retirement in Europe (SHARE). Temporal associations were obtained using generalized estimating equations. All models were controlled for prior sociodemographic, personality, lifestyle factors, health behaviors, and pre-baseline leisure activity values and all outcome variables. The Bonferroni correction was used to correct for multiple testing. E-values were calculated to examine the sensitivity of the associations to unmeasured confounding. Secondary analyses (1) under the complete case scenario, (2) after excluding respondents with health conditions, and (3) using a limited set of covariates were conducted to provide evidence for the robustness of the results.

**Results:**

The relaxed solitary activity of reading almost daily was prospectively associated with a lower risk of depression, experiencing pain, daily functioning limitations, cognitive impairment, lower loneliness scores, and more favorable wellbeing outcomes. Engaging in serious solitary leisure activities almost daily was prospectively associated with a lower risk of depression, feeling full of energy, and a lower risk of death by any cause. Occasionally engaging in these activities was prospectively associated with greater optimism and a lower risk of cognitive impairment. Engaging in serious social activities was prospectively associated with greater happiness, lower scores on the loneliness scale, a lower risk of Alzheimer's disease, and an increased risk of cancer. Additionally, occasionally engaging in serious social activities was associated with greater optimism and lower risk of depression, pain, and mobility limitations. These associations were independent of demographics, socioeconomic status, personality, history of diseases, and prior lifestyle. The sensitivity analyses provided substantial evidence for the robustness of these associations.

**Discussion:**

Mind-engaging leisure activities can be considered a health and wellbeing resource. Practitioners may consider them tools that help middle-aged and older adults maintain their health and quality of life.

## 1. Introduction

Humans seek leisure. It can be considered a complex human need with various adaptive purposes (1). These include improving physiological and psychological functioning (2), enjoying entertainment and pleasant moments (3), seeking distraction from daily concerns and stressful work (4), spending time with family, friends, and acquaintances (5, 6), coping with social crises (7), and even informal learning (8). In addition, leisure seems to constitute one of the most fundamental human behaviors and a part of our daily routines since the early stages of human evolution (9, 10).

The concept of leisure comprises a set of heterogeneous activities that can differ in terms of the intensity of physical activity, degree of sociability, and level of intellectual effort (11–13). Special attention has been paid to the role of leisure studies in improving and maintaining good health and wellbeing (13–18). Various leisure activities have been systematically investigated concerning their salutogenic effects. For example, prior studies have shown that arts and cultural activities may reduce the allostatic load by reducing unhealthy habits such as smoking and alcohol consumption (19) and directly improve hedonic wellbeing (17). Leisure activities that require physical exercise reduce the risk of cardiovascular mortality regardless of age, sex, and pre-existing diseases (20, 21). Social leisure activities improve emotional wellbeing and reduce depression and anxiety symptoms (17), especially among socially deprived participants (22). Despite numerous other examples, the pathways through which various leisure activities nurture beneficial effects on health and wellbeing remain unclear. Fancourt et al. (13) provided the first systematic survey of the types of effects and possible pathways, indicating that much more research is needed to understand the actual mechanisms thoroughly.

Therefore, it is necessary to examine the potential health and wellbeing benefits associated with different forms of leisure that have not received sufficient scientific attention so far. Consequently, the present study considers a particular class of leisure activities, namely, mind-engaging leisure activities. These may include reading, word and number games, playing cards or chess, or similar activities. One may question this choice of leisure activities arguing that mind games are merely sedentary activities, which, as part of sedentary lifestyles, have contributed to the worsening of population health and led to adverse health outcomes (23–25). However, in addition to the well-known positive role of physical exercise on health and prevention of chronic diseases (26–29) and cognitive functioning in older age (30), sedentary activities that keep the mind engaged have also been praised in similar contexts (31).

Regardless, evidence of the effects of various types of mind-stimulating leisure activities on health remains limited, with some preliminary results indicating a positive impact on mental health (32). Consequently, we examined three categories of cognitive leisure activities that involve brain exercises and mind games in this study: (1) doing word or number games such as crossword puzzles or Sudoku; (2) playing cards or games such as chess; and (3) reading books, magazines, and newspapers. These activities reflect the classical partitioning of leisure activities into relaxed, serious, and social (13, 33). Serious leisure activities are usually problem-solving-oriented and cognitively engaging mind games. Relaxed leisure activities are non-problem-solving-oriented, although they can still be cognitively engaging. Among these two groups, we can further distinguish between solitary and social forms of activity.

Consequently, we classify word or number games—such as crossword puzzles or Sudoku—as serious and solitary leisure activities. Playing social games such as cards or chess is classified as a serious and social leisure activity. Lastly, reading books, magazines, or newspapers is classified as a relaxed and solitary leisure activity. The first two types of activities involve mind games comprising directed cognitive effort, with the former being solitary and the latter involving a social component.

Reading is engaging, and emotionally and cognitively arousing. However, the reader has no specific goal, contrary to the gamer who wants to win. This makes reading an especially interesting example of a relaxing activity in which one pursues narrative immersion, enjoyment, information gathering, and more, without any specific performance check. Instead, mind games generate a serious dimension of experience that relates to testing one's intellectual abilities on a particular problem that may or may not be solved, which can be engaging and pleasant but not necessarily relaxing. In solitary mind games, the performance check is individual, while in social mind games it is relational, resulting in competition with others. The comparison of these three types of activities with their peculiarities in terms of their effects on health and wellbeing has, to our knowledge, not been explored in the literature before. Therefore, it is essential to understand the detailed mechanisms through which certain leisure activities may benefit individuals undertaking them.

This study examined prospective associations between the frequency of the three mind-stimulating leisure activities and physical health, emotional wellbeing, cognitive impairment, daily life functioning outcomes, and all-cause mortality. We also addressed two research questions: First, what changes in physical health, emotional wellbeing, cognitive impairment, and daily life functioning outcomes could be observed within 6 years, if people engage in any of the three examined leisure activities classes? Second, what changes in longevity may be observed when people engage in any of the leisure activities? We were particularly interested in studying these effects in middle-aged and older adults.

## 2. Materials and methods

### 2.1. Study population

Longitudinal data from the Survey of Health, Ageing, and Retirement in Europe (SHARE) (34) were used. Detailed information regarding the data sources is provided in the Supplementary material. This study used data from 2011 to 2020. The analytical sample included middle-aged and older adults aged 50 years or older who participated in Waves 4, 5, and 8. A total of 19,821 participants met the inclusion criteria. In the analysis of all-cause mortality, 39,009 middle-aged and older adults who participated in Waves 4 and 5 were observed for up to 8 years. They were from 15 European countries: Austria, Germany, Sweden, the Netherlands, Spain, Italy, France, Denmark, Switzerland, Belgium, Czech Republic, Poland, Hungary, Slovenia, and Estonia.

### 2.2. Measures

#### 2.2.1. Leisure activities

The relaxed solitary leisure activity—reading—was assessed by asking respondents about the frequency with which they read books, magazines, and newspapers (almost every day, sometimes, and never). The frequency of serious solitary leisure activities involving playing word or number games, such as crossword puzzles or Sudoku, was also examined (almost every day, sometimes, and never). The frequency of serious social activity involving playing cards or games such as chess was also considered (almost every day, sometimes, and never).

#### 2.2.2. Outcomes

Health and wellbeing outcomes such as (1) a sense of loneliness; (2) depression; (3) self-reported diagnosis of Alzheimer's disease, dementia, or other serious memory impairment; (4) sense of meaning in life; (5) happiness; (6) feeling energetic; and (7) optimism. We examined daily life functioning using instruments measuring the level of difficulty in activities of daily living (ADL) and instrumental activities of daily living (IADL) due to physical, mental, emotional, and memory problems. The following physical health outcomes were examined: heart attack, hypertension, high blood cholesterol, stroke, diabetes, and cancer. We accounted for both non-fatal and fatal conditions. We also considered the presence of impaired pain and whether the respondents experienced at least one mobility, arm, or fine-motor limitation. Cognitive impairment was assessed using a measure of time orientation. All-cause mortality was also considered an outcome. Detailed information regarding the measures used is presented in the Supplementary material.

#### 2.2.3. Covariates

All covariates were self-reported and measured during the pre-baseline (Wave 4). These included demographic characteristics (gender, age, marital status, education, and country), socioeconomic factors (income and wealth), personality traits (agreeableness, openness, neuroticism, conscientiousness, and extraversion), health behaviors (sports activity, alcohol consumption, and BMI), and lifestyle factors (volunteering). Detailed information regarding the covariates used is presented in the Supplementary material.

#### 2.2.4. Prior values of outcomes and exposure

To reduce the possibility of reverse causation and residual confounding, we adjusted for the prior values of the 21 outcome variables (i.e., prior emotional wellbeing, daily life functioning, cognitive impairment, and history of diseases). To further reduce the risk of reverse causality, we controlled for prior values of the respective exposure variable (i.e., mind-stimulating leisure activity).

### 2.3. Statistical analysis

We conducted an outcome-wide analysis (35). In this analysis, 21 outcomes were used to extensively examine the pattern of temporal associations with leisure activities (at a 6-year follow-up). Previous studies have shown this methodology to be useful in limiting the risk of preferring only significant results and salami-slicing, as well as revealing patterns of associations that may not be apparent if a single outcome was examined (36–40). Each prospective association was modeled using generalized estimating equations. We clustered by country and adjusted standard errors to account for the hierarchical nature of the data. Three types of estimates are reported. Standardized regression estimates were used for continuous outcomes. For dichotomous outcomes, odds ratios for rare outcomes (i.e., occurring in <10% of the population) and risk ratios for non-rare outcomes (i.e., occurring in at least 10% of the population) were indicated. Poisson regression with robust standard errors was used to estimate risk ratios (41, 42), and the Bonferroni correction was used to correct for multiple testing. All missing covariates, exposure, and outcome variables were imputed using chained equations [10 sets of imputed data were generated (43)] and multiple imputation estimates were pooled using the Rubin rule (44).

A series of robustness checks was carried out. First, the robustness of the results was examined using E-values. This sensitivity measure assesses the extent to which a potential uncontrolled confounder would need to be associated with both the exposure and outcome to explain the observed association (45). Second, all models were rerun after excluding anyone with a history of a given physical condition at the pre-baseline. Third, the primary set of models was reanalyzed using complete case analysis to assess the robustness of the results to missing data patterns. Finally, the primary models were rerun with a limited set of controls (i.e., only demographic and socioeconomic control variables traditionally used in similar analyses), as there was a risk that with such an extensive set of covariates, the models could have been overfitted. All statistical analyses were performed using Stata/SE 17.0.

## 3. Results

### 3.1. Descriptive analyses

In the pre-baseline Wave (Wave 4), participants were, on average, 64.5 (SD = 8.72) years old, mostly women (59.3%) and married (69.6%), with upper secondary (36.1%) or first-stage tertiary education (22.2%). More than 66% of participants reported reading books, magazines, and newspapers almost daily, 12.2% sometimes, and 21.3% never. More than 25% of middle-aged and older adults engaged with number and word games daily, 21.6% sometimes, and 52.9% never. 4.3% of participants played cards or other games daily, 27.2% sometimes, and 68.6% never. Table 1 shows the participants' characteristics at pre-baseline. Table 2 shows the distribution of health and wellbeing outcomes. Supplementary Table 1 presents the participant characteristics at pre-baseline for each leisure activity.

**Table 1:** Distribution of participant characteristics at the study prebaseline wave (wave 4, *N* = 19,821).

**Participant characteristic**	**Total (*****N*** = **19,821)**	
	**%**	**Mean (SD)**
**Sociodemographic factors**		
Gender–Male	40.71	
**Age group**		
50–59	31.06	
60–69	40.07	
70–79	23.36	
80+	5.52	
**Marital status**		
Married and living together with the spouse	69.61	
Registered partnership	1.48	
Married but living separately	1.32	
Never married	5.53	
Divorced	9.65	
Widowed	12.41	
**Education attainment (ISCED-97)**		
None	2.27	
Primary education or first stage of basic education	15.04	
Lower secondary or second stage of basic education	17.95	
(Upper) secondary education	36.13	
Post-secondary non-tertiary education	5.55	
First stage of tertiary education	22.22	
Second stage of tertiary education	0.84	
Annual personal income (Euro)		32,041 (48,026)
Household net financial assets (Euro)		60,746 (192,095)
**Country**		
Austria	7.16	
Germany	3.28	
Sweden	4.32	
The Netherlands	5.30	
Spain	6.04	
Italy	5.71	
France	10.72	
Denmark	5.29	
Switzerland	9.05	
Belgium	6.89	
Czech Republic	10.15	
Poland	3.13	
Hungary	3.74	
Slovenia	5.84	
Estonia	13.38	
**Personality traits**		
Extraversion (1–5)		3.51 (0.94)
Agreeableness (1–5)		3.70 (0.80)
Consciousness (1–5)		4.11 (0.79)
Neuroticism (1–5)		2.58 (1.01)
Openness (1–5)		3.37 (0.97)
**Lifestyle factors**		
BMI		27.03 (4.75)
Volunteering or charity work (yes)	19.41	
**Alcohol consumption**		
Almost every day	17.44	
5-6 days a week	2.99	
3-4 days a week	7.45	
Once or twice a week	19.57	
Once or twice a month	13.12	
Less than once a month	11.74	
Not at all in the last 6 months	27.69	
**Sport activity requiring a moderate level of energy**		
More than once a week	73.24	
Once a week	13.38	
One to three times a month	5.39	
Hardly ever or never	7.99	

Survey of Health, Ageing and Retirement in Europe (SHARE), Middle-aged and older adults aged 50 years and older.

**Table 2:** Study outcomes at the study prebaseline wave (wave 4, *N* = 19,821).

**Study outcomes**	**Total (*****N*** = **19,821)**	
	**%**	**Mean (SD)**
**Wellbeing**		
Loneliness (3-item loneliness scale; 3–9)		3.37 (0.97)
Alzheimer's disease	0.51	
Depression (EURO-D≥4)	25.28	
Future looks good (1–4)		3.12 (0.90)
I feel full of energy these days (1–4)		3.26 (0.82)
On balance, I look back on my life with a sense of happiness (1–4)		3.39 (0.77)
I look forward to each day (1–4)		3.49 (0.83)
I feel that my life has meaning (1–4)		3.61 (0.69)
**Daily life functioning**		
ADL (at least 1 limitation)	7.60	
IADL (at least 1 limitation)	12.31	
**Physical health**		
Heart attack	10.98	
Hypertension	38.49	
High blood cholesterol	22.55	
Stroke	2.82	
Diabetes	10.59	
Cancer	4.12	
Pain	42.55	
Mobility (at least 1 limitation)	45.66	
**Cognitive Impairment**		
Date orientation (0–4)		3.85 (0.52)

Survey of Health, Ageing and Retirement in Europe (SHARE), middle-aged and older adults aged 50 years and older.

### 3.2. Relaxed leisure activity—reading books, magazines, and newspapers and subsequent wellbeing, physical health, daily life functioning, cognitive impairment, and all-cause mortality

At the 6-year follow-up, middle-aged and older adults who read books, magazines, and newspapers almost every day had a substantially lower risk of being diagnosed with depression (by 7% compared to respondents who did not read at all; 95% CI = 0.904, 0.949, *p* < 0.001) (Table 3). They also scored lower on the loneliness scale (β = −0.056, 95% CI = −0.111, −0.001, *p* = 0.045). Compared to those who did not read at all, they reported higher scores in several wellbeing dimensions, such as “Future looks good” (β = 0.097, 95% CI = 0.047, 0.147, *p* = 0.003), “I feel full of energy these days” (β = 0.085, 95% CI = 0.027, 0.144, *p* = 0.010), “On balance, I look back at my life with a sense of happiness” (β = 0.135, 95% CI = 0.084, 0.186, *p* < 0.001), “I look forward to each day” (β = 0.097, 95% CI = 0.041, 0.153, *p* = 0.003), and “I feel that my life has meaning” (β = 0.107, 95% CI = 0.056, 0.159, *p* = 0.001). Additionally, they had a substantially lower risk of limitations in ADL (RR = 0.880, 95% CI = 0.797–0.973, *p* = 0.012) and IADL (RR = 0.913, 95% CI = 0.863, 0.966, *p* = 0.002), as well as a lower risk of chronic and impaired pain (RR = 0.948, 95% CI = 0.903, 0.996, *p* = 0.033). Finally, they scored higher on the time-orientation scale (β = −0.077, 95% CI = 0.014, 0.139, *p* = 0.021). These temporal associations were independent of demographic and socioeconomic status, personality, health history, previous daily life functioning, health behaviors, and lifestyle. They were also independent of their history of reading books, magazines, and newspapers.

**Table 3:** Prospective associations between reading books, magazines, or newspapers, doing word and number games and playing cards or games, and wellbeing, physical health, daily life functioning, cognitive impairment, and all-cause mortality^a^.

		**Reading books, magazines, and newspapers (ref**. = **never)**		**Doing word or number games (ref**. = **never)**		**Playing cards or games (ref**. = **never)**	
**Outcome**	**Statistics**^b^, ^c^	**Sometimes** ^d^	**Almost every day**	**Sometimes** ^d^	**Almost every day**	**Sometimes** ^d^	**Almost every day**
**Emotional wellbeing**							
Loneliness (3-item loneliness scale)	β^b^ (95% CI) *p*-value	−0.035 (−0.094, 0.024) 0.215	−0.056^†^ (−0.111, −0.001) 0.045	−0.025 (−0.068, 0.018) 0.228	−0.029 (−0.085, 0.026) 0.265	−0.060 (−0.090, −0.031) 0.002	−0.097 (−0.142, −0.051) 0.001
Alzheimer's disease	OR (95% CI) *p*-value	0.880 (0.611, 1.269) 0.492	0.696 (0.461, 1.051) 0.085	0.822 (0.609, 1.109) 0.199	0.834 (0.629, 1.106) 0.206	0.829 (0.628, 1.094) 0.182	0.606^†^ (0.415, 0.884) 0.009
Depression (EURO-D≥4)	RR (95% CI) *p*-value	0.969 (0.909, 1.032) 0.313	0.925^†^ (0.861, 0.994) 0.035	0.921^†^ (0.863, 0.983) 0.013	0.927^†^ (0.864, 0.994) 0.034	0.904^†^ (0.844, 0.969) 0.005	0.936 (0.844, 1.039) 0.216
Future looks good	β^b^ (95% CI) *p*-value	0.067^†^ (0.023, 0.111) 0.008	0.097^†^ (0.047, 0.147) 0.003	0.048^†^ (0.002, 0.095) 0.041	0.056 (−0.002, 0.114) 0.055	0.046^†^ (0.008, 0.083) 0.022	0.024 (−0.034, 0.082) 0.384
I feel full of energy these days	β^b^ (95% CI) *p*-value	0.038 (−0.011, 0.087) 0.114	0.085^†^ (0.027, 0.144) 0.010	0.041^†^ (0.008, 0.073) 0.020	0.054^†^ (0.012, 0.095) 0.018	0.022 (−0.014, 0.058) 0.204	−0.014 (−0.080, 0.052) 0.641
On balance, I look back on my life with a sense of happiness	β^b^ (95% CI) *p*-value	0.069 (−0.001, 0.140) 0.053	0.135 (0.084, 0.186) < 0.001	0.019 (−0.014, 0.052) 0.226	0.026 (−0.034, 0.085) 0.361	0.047 (−0.006, 0.100) 0.074	0.082^†^ (0.023, 0.141) 0.010
I look forward to each day	β^b^ (95% CI) *p*-value	0.076^†^ (0.007, 0.144) 0.033	0.097^†^ (0.041, 0.153) 0.003	0.066 (0.034, 0.098) 0.001	0.049 (−0.001, 0.099) 0.053	0.070^†^ (0.031, 0.110) 0.003	0.019 (−0.053, 0.091) 0.570
I feel that my life has meaning	β^b^ (95% CI) *p*-value	0.045 (−0.021, 0.110) 0.153	0.107 (0.056, 0.159) 0.001	0.026 (−0.002, 0.055) 0.068	0.016 (−0.023, 0.055) 0.388	0.037 (−0.012, 0.086) 0.125	−0.015 (−0.105, 0.075) 0.718
**Daily life functioning**							
ADL (at least 1 limitation)	RR (95% CI) *p*-value	0.926 (0.844, 1.017) 0.107	0.880^†^ (0.797, 0.973) 0.012	0.948 (0.854, 1.052) 0.317	0.940 (0.837, 1.055) 0.291	0.965 (0.878, 1.059) 0.449	1.043 (0.911, 1.193) 0.542
IADL (at least 1 limitation)	RR (95% CI) *p*-value	0.909^†^ (0.836, 0.989) 0.026	0.913 (0.863, 0.966) 0.002	0.940 (0.866, 1.019) 0.134	0.924 (0.827, 1.032) 0.163	0.960 (0.880, 1.046) 0.351	1.012 (0.887, 1.156) 0.857
**Physical health**							
Heart attack	OR (95% CI) *p*-value	0.823 (0.645, 1.050) 0.114	1.069 (0.892, 1.282) 0.468	0.944 (0.859, 1.037) 0.228	1.009 (0.916, 1.111) 0.857	0.999 (0.913, 1.093) 0.987	0.945 (0.749, 1.197) 0.647
Hypertension	RR (95% CI) *p*-value	0.976 (0.913, 1.043) 0.471	0.973 (0.918, 1.032) 0.356	1.005 (0.963, 1.050) 0.807	0.980 (0.944, 1.017) 0.288	1.007 (0.963, 1.054) 0.746	1.006 (0.902, 1.129) 0.920
High blood cholesterol	RR (95% CI) *p*-value	0.994 (0.912, 1.084) 0.894	0.957 (0.874, 1.048) 0.339	1.001 (0.939, 1.066) 0.984	1.021 (0.949, 1.098) 0.582	1.036 (0.980, 1.095) 0.210	1.025 (0.925, 1.135) 0.638
Stroke	OR (95% CI) *p*-value	0.849 (0.628, 1.147) 0.286	0.810 (0.627, 1.046) 0.106	0.910 (0.725, 1.134) 0.388	0.881 (0.667, 1.163) 0.370	1.196 (0.965, 1.481) 0.101	1.192 (0.821, 1.731) 0.356
Diabetes	RR (95% CI) *p*-value	0.968 (0.867, 1.081) 0.561	1.070 (0.982, 1.166) 0.123	0.978 (0.917, 1.044) 0.510	1.002 (0.926, 1.083) 0.966	0.970 (0.893, 1.053) 0.464	0.970 (0.880, 1.070) 0.548
Cancer	RR (95% CI) *p*-value	0.950 (0.840, 1.075) 0.417	1.006 (0.900, 1.125) 0.914	0.932 (0.754, 1.152) 0.514	0.955 (0.790, 1.153) 0.629	1.212^†^ (1.014, 1.447) 0.034	1.456^†^ (1.074, 1.973) 0.016
**Outcome**	**Statistics**^b^, ^c^	**Sometimes** ^d^	**Almost every day**	**Sometimes** ^d^	**Almost every day**	**Sometimes** ^d^	**Almost every day**
Pain	RR (95% CI) *p*-value	0.990 (0.941, 1.041) 0.688	0.948^†^ (0.903, 0.996) 0.033	0.979 (0.940, 1.019) 0.292	0.968 (0.910, 1.029) 0.296	0.947^†^ (0.912, 0.983) 0.004	0.994 (0.921, 1.072) 0.871
Mobility	RR (95% CI) *p*-value	1.011 (0.982, 1.041) 0.456	0.982 (0.951, 1.013) 0.250	1.004 (0.961, 1.048) 0.872	0.970 (0.937, 1.003) 0.075	0.960^†^ (0.933, 0.988) 0.005	1.011 (0.965, 1.060) 0.642
**Cognitive impairment**							
Date orientation	β^b^ (95% CI) *p*-value	0.075^†^ (0.003, 0.147) 0.043	0.077^†^ (0.014, 0.139) 0.021	0.052^†^ (0.008, 0.095) 0.024	0.026 (−0.018, 0.070) 0.224	0.034 (−0.007, 0.075) 0.095	0.049 (−0.014, 0.112) 0.111
All-cause mortality	OR (95% CI) *p*-value	0.903 (0.792, 1.031) 0.132	0.910 (0.822, 1.007) 0.068	0.865^†^ (0.748, 0.999) 0.049	0.867^†^ (0.785, 0.956) 0.004	0.938 (0.813, 1.082) 0.378	0.896 (0.727, 1.104) 0.301

CI, confidence interval; OR, odds ratio; RR, risk ratio; ADL, activities of daily living; IADL, instrumental activities of daily living.

a Missing covariate variables were imputed using chained equations (ten sets of imputed data were generated). All models were controlled for participant demographics: age, gender, marital status, educational attainment, and country; socioeconomic factors: annual personal income, household net financial assets, health behaviors such as BMI, alcohol consumption, and sports activity; lifestyle factors demonstrated in volunteer activities; and personality traits such as agreeableness, openness, conscientiousness, neuroticism, and extraversion. Each model was also adjusted for prior values of the 21 outcome variables and of the exposure variable, as well as previous self-reported presence/absence of diagnosis for heart attack, hypertension, high blood cholesterol, stroke, diabetes, and cancer simultaneously in each regression model.

b All continuous outcomes were standardized (mean = 0, standard deviation = 1), and β was the standardized effect size.

c p < 0.05 after Bonferroni correction (p-value cut-off for Bonferroni correction = 0.05/21 outcomes = 0.0024).

d Sometimes comprises almost every week, almost every month, less often.

† Not significant after Bonferroni.

Survey of Health, Ageing and Retirement in Europe (SHARE), adults 50 years and older (N = 19,821).

There was no evidence that reading books, magazines, or newspapers was prospectively associated with all-cause mortality and physical health outcomes, including heart attack, hypertension, high blood cholesterol, stroke, diabetes, and cancer (at a 6-year follow-up).

### 3.3. Serious and solitary mind games (number and word games) and subsequent wellbeing, physical health, daily life functioning, cognitive impairment, and all-cause mortality

Middle-aged and older adults who engaged in serious solitary mind games (number and word games) almost daily had a 7% lower risk of depression (RR = 0.927, 95% CI = 0.864, 0.994, *p* = 0.034) and a 13% lower risk of death regardless of the cause of death (RR = 0.867, 95% CI = 0.785, 0.956, *p* = 0.004) compared to middle-aged and older adults who did not engage in these activities (Table 3). They also reported higher scores for feeling full of energy (β = 0.054, 95% CI = 0.012, 0.095, *p* = 0.018) than those who did not engage in the activity. Regarding optimism reflected in looking forward to each day (“I look forward to each day”) and a positive future outlook (“Future looks good”), middle-aged and older adults who sometimes engage in number and word games scored higher than those who did not engage (β = 0.066, 95% CI = 0.034, 0.098, *p* = 0.001; β = 0.048, 95% CI = 0.002, 0.095, *p* = 0.041, respectively). However, the effects for respondents who engage almost daily are similar in size to those engaging occasionally but not significant due to wider confidence intervals. Similarly, we found that better time orientation was associated with prior occasional (i.e., sometimes) engagement in number and word games but not everyday engagement (β = 0.052, 95% CI = 0.008, 0.095, *p* = 0.024).

The associations were independent of demographic and socioeconomic status, personality, health history, prior daily life functioning, health behaviors, lifestyle, and previous engagement in word and number games. No prospective associations were found between engagement in word and number games, indicators of daily life functioning, and physical health outcomes.

### 3.4. Serious and social mind games (playing cards and games) and subsequent wellbeing, physical health, daily life functioning, cognitive impairment, and all-cause mortality

Playing cards and games almost daily were prospectively associated with lower scores on the loneliness scale (β = −0.097, 95% CI = −0.142, −0.051, *p* = 0.001), a 39% lower risk of Alzheimer's disease (OR = 0.606, 95% CI = 0.415, 0.884, *p* = 0.009), and a 56% increased risk of cancer (RR = 1.456, 95% CI = 1.074, 1.973, *p* = 0.016) compared with those who did not perform the activities (Table 3). Additionally, it was found that playing cards and games only sometimes compared to not at all was associated with an increased optimism (“Future looks good”, β = 0.046, 95% CI = 0.008, 0.083, *p* = 0.022; “I look forward to each day,” β = 0.070, 95% CI = 0.031, 0.110, *p* = 0.003). Furthermore, middle-aged and older adults who occasionally engaged in this activity had a 10% lower risk of depression (RR = 0.904, 95% CI = 0.844, 0.969, *p* = 0.005), a 5% lower risk of feeling impaired or chronic pain (RR = 0.947, 95% CI = 0.912, 0.983, *p* = 0.004), and a 4% lower risk of experiencing mobility limitation (RR = 0.960, 95% CI = 0.933, 0.988, *p* = 0.005).

The associations were independent of demographic and socioeconomic status, personality, health history, prior daily life functioning, health behaviors, lifestyle, and engagement in playing cards and games. No prospective associations were found between engagement in activities involving playing cards and games, quality of life indicators, physical health outcomes, or all-cause mortality.

### 3.5. Robustness analysis

When rerunning the models after excluding respondents with a pre-baseline health condition (i.e., depression, heart attack, hypertension, high blood cholesterol, stroke, diabetes, cancer, and Alzheimer's disease), most of the prospective associations examined remained significant but sometimes slightly attenuated (Supplementary Table 2). In the complete case scenario, the results were similar to those obtained from the primary analyses of the imputed dataset (Supplementary Table 3). Additionally, the analyses rerun with the limited set of covariates (Supplementary Table 4) yielded very similar results to those obtained from the primary analyses; however, the effect sizes were larger and several non-significant associations from the primary analyses became significant. This provides additional evidence supporting the robustness of temporal associations between leisure activities and all-cause mortality, wellbeing, physical health, daily life functioning, and cognitive impairment outcomes.

The E-values suggest that the observed associations were modestly robust to unmeasured confounding factors (Supplementary Table 5). The most robust associations (the largest E-value) were those between serious and social mind games, that is, participation in activities involving playing cards and games, and two diseases: Alzheimer's disease and cancer.

## 4. Discussion

This study examined the temporal associations between engaging in three specific classes of mind-engaging leisure activities and 21 subsequent outcomes. The results indicate that different forms of mind-engaging leisure have distinctively different effects on middle-aged and older adults. This supports the idea that a specific focus on the type of activity carried out is crucial when evaluating its health and wellbeing benefits.

We found particular differences between reading and problem-solving leisure activities (mind games), whether solitary or social, which may be due to the variable natures of these activities. In the case of reading, the focus is on meaning and content, whereas in the case of mind games, it is on performance. Cognitive resources are always engaged but under different conditions and with different patterns and goals. In experiencing meaning through reading, participants explore possibilities (whether real or fictional) and absorb information. In doing mind games, they look for solutions to specific problems. Moreover, while engaging in solitary mind games, one's cognitive resources are tested; in social mind games, such resources are also compared to the resources of others with the additional implication of social rewards (i.e., winning vs. losing). In addition, some social mind games, such as poker, may be potentially addictive (46). Therefore, the corresponding neural pathways that supersede these activities may differ (47–50).

These differences are reflected in our results, where specific effect patterns were observed for each leisure activity class. For reading, there is a clear and strong association with wellbeing, corroborating previous findings of the structured review of 12 studies by Latchem and Greenhalgh (18). There is also a significant effect of reading on depressive symptoms, which, despite being inconsistent with the findings for middle-aged and older adults in the US reported by Bone et al. (51), corroborates the findings of a randomized controlled study by Kaltenegger et al. (52). Additionally, the prospective association between reading and subsequent reduced chronic pain and better daily life functioning corroborates previous limited findings (52, 53). Similar to previous studies, there is also a positive cognitive effect regarding time orientation (54). Additionally, reading had a positive effect on reduced loneliness, which corroborates previous qualitative and quantitative findings in other studies (55–58). This could seem counterintuitive as reading is generally a solitary activity, however, it is important to realize that, at least for certain types of reading activities such as reading fiction, an important dimension of simulation of social interaction is essentially involved (59). This may positively affect perception of loneliness, as the reader is immersed in social situations and may even develop an identification with fictional characters (57) and a better real-life ability to empathize (58).

We found an association between regular and frequent reading of books, magazines, and newspapers (almost daily) and increased subsequent sense of meaning in life. This result extends the list of antecedents of meaning in life, adding this activity to previously documented purpose and meaning determinants. These determinants include positive affect, social connections, orientation to promote good, feeling purposeful at work, wealth, and income (37, 39, 60–62). The reading experience appears to unlock a wide spectrum of benefits. They were mostly concentrated in the psycho-socio-behavioral sphere, as no significant effects on physical health outcomes and mortality were found. Furthermore, a positive and linear association was found between exposure to reading activities and health effects.

Solitary mind games provide different sets of benefits. We observed a positive effect on depressive symptoms and a lower risk of mortality. There was also a positive effect on a specific interoceptive wellbeing dimension (feeling full of energy). However, other wellbeing dimensions, such as optimism, were positively affected only when the activity was occasional and not regular. When it was regular, the effect was similar in size but not significant due to wider confidence intervals. Notably, for cognitive impairment. Occasional engagement was found to be more beneficial than regular engagement. This may seem counterintuitive to the idea that cognitive function is maintained through constant exercise and that mind games are the mental equivalent of gym activity to benefit physical fitness (20, 26, 27). Although our findings do not present a clear picture of the associations examined, the complexity of the associations is in line with previous studies, which reported mixed results. On the one hand, there is evidence on the lack of positive impact of cognitive brain training on the cognition and wellbeing of working adults (63). On the other hand, the effect of (1) inductive reasoning training on decreased difficulty with instrumental activities of daily living (IADL) (64) and (2) verbal episodic memory, inductive reasoning, and speed of processing trainings on cognitive abilities among older adults (64), are presented.

Although it is difficult to interpret these results clearly, we can observe that regular engagement in solitary mind games does not entail any form of simulated social interaction (contrary to reading). Therefore, an excessive focus on these activities might have a negative trade-off with other social activities that have a complementary impact on emotional wellbeing. Furthermore, the solitary cognitive function exercise may have an excessively narrow focus to truly preserve cognitive fluency considering the importance of interaction-related cognition (including collective thinking) in humans. Therefore, individualistic and meaningless exertion of cognitive function for the preservation of cognitive fluency may not be optimal in the medium-long term (65). Unlike reading, regular solitary mind games may have an important positive effect on mortality risk, which requires further analysis. Compared to reading, the effects of solitary mind games are narrower in scope, related to moderate rather than regular exposure, and more evenly split between the psycho-socio-behavioral, physical health, and longevity dimensions. Contrary to previous findings (64), no prospective associations with daily functioning (ADL and IADL) were found.

Social-mind games are altogether different. Similar to previous studies (56, 66), the positive effects of regular exposure to social mind games impact loneliness (as expected) and mobility limitations. However, there is also a significantly higher risk of cancer and a reduced risk of Alzheimer's disease. This finding on the risk of cancer warrants further research. However, we can only hypothesize that this social activity might have been concurrent with unfavorable health behaviors, such as smoking or drinking alcohol, well-known cancer risk factors (67). Social mind games, similar to solitary games, generate certain positive effects only if they are performed through limited exposure. This is the case for optimism and hedonic wellbeing, pain perception, and depression. Concerning social mind games, there seems to be an upper limit beyond which further activity is less beneficial for certain outcomes. In this case, it cannot be linked to a lack of sociality, as these activities are inherently social; rather, it is related to the specific conditions of such sociality. In particular, stress could be the negative component typically associated with competitive situations (68). Regular participation in competitive games could lead to permanent stressful arousal that, in the medium-long term, could be detrimental for wellbeing and even health, particularly for increased cancer risk. This hypothesis is consistent with previous research documenting the detrimental role of stress in wellbeing (69, 70) and the onset of disease (71, 72), such as cancer (73–75). Less regular exposure seems to be beneficial, as stressful arousal is limited, and possibly stimulating for aging participants. Meaning-oriented forms of social interaction less related to stressful arousal are likely to have different effects. However, our results on the prospective association between participation in social mind games and the reduced risk of Alzheimer's disease corroborate previous evidence linking cognitive (leisure) activities with this disease and other dementias (76–78). However, our findings indicate that not only simply exercising cognitive function may be beneficial, but also the social component inherent in the activities may be important.

Unlike reading, mind games have issues of excess exposure and a narrower range of benefits, including some crucial risks in the case of social mind games. Purely exercising cognitive function in solitary and social contexts may be beneficial, but this should not involve too much time or too many mental resources. Instead, activities that are cognitively engaging but related to meaning, even if solitary, can generate a wide range of benefits and positively affect loneliness.

### 4.1. Strengths and limitations

This study was based on a large sample and benefitted from a longitudinal design. This made it possible to make inferences about prospective associations and to account for a wide set of confounders. We found several effects, some of which could be deemed intuitive. In contrast, others are notable and encourage further research with implications beyond the scope of the present study. These associations and effects were independent of demographic and socioeconomic status, personality, lifestyle, health behaviors, and medical history. These temporal associations were also reasonably robust to unmeasured confounding, missing data patterns, and medical history (considering only new instances of disease vs. controlling for the history of diseases).

The study's main limitation is that we could not directly test the possible pathways behind the effects we found. For this purpose, we would need biobehavioral measurements and indicators that, to our knowledge, are rarely collected and not yet fully developed from a conceptual and methodological viewpoint. Therefore, we hope this study will increase interest in this new and promising research direction. Next, this study used self-reported data on health conditions, wellbeing, and daily life functioning outcomes, which makes our results subject to social desirability bias. However, there is some reassurance that this bias did not negatively affect the accuracy of the results owing to the longitudinal design and control of pre-baseline outcomes and exposure. Our use of self-reported health outcomes may also have influenced the accuracy of the results. However, previous studies provide some confidence in this regard, as they report a high agreement between medical records and self-reported disease data (79). Finally, the data used were collected from middle-aged and older adults, which might reduce the accuracy and generalizability of our results. However, most of the instruments used were developed specifically for this population. Nevertheless, further studies are required to corroborate our findings in different populations.

## 5. Conclusions

Leisure is a very broad category of activities, and their effects on health and wellbeing can be very different as shown in prior studies (13, 16, 17, 80–84). In this paper, we present a case of differences in health and wellbeing impacts across relatively similar cognitive leisure activities. These are sedentary forms of leisure, such as reading books, magazines, and newspapers; doing word and number games (solitary mind games); and playing cards and other competitive games, such as chess (social mind games). The fact that the experience—individual or social—involves cognitive stimulation related to meaning rather than pure cognitive performance has an important difference in its effects on wellbeing and health outcomes. This implies that exploring the differences in the biobehavioral pathways that cause such diverse outcomes is important to gain a deeper understanding of these effects.

We enter a new research stage on the health and wellbeing effects of cognitive leisure activities. The main issue is no longer whether leisure may have such effects but rather how and why such effects occur. By posing these questions, we can design more targeted and possibly effective policy interventions. Such interventions will especially benefit fragile and disadvantaged participants, such as older adults. Moreover, they may benefit others who are generally disadvantaged in terms of wellbeing and health opportunities; this includes those who are socioeconomically deprived, marginalized, and with important psychological and medical conditions.

## Data availability statement

Publicly available datasets were analyzed in this study. This data can be found at: (1) Börsch-Supan, A. (2022). Survey of Health, Ageing and Retirement in Europe (SHARE) Wave 4. Release version: 8.0.0. SHARE-ERIC. Data set. doi: 10.6103/SHARE.w4.800. (2) Börsch-Supan, A. (2022). Survey of Health, Ageing and Retirement in Europe (SHARE) Wave 5. Release version: 8.0.0. SHARE-ERIC. Data set. doi: 10.6103/SHARE.w5.800. (3) Börsch-Supan, A. (2022). Survey of Health, Ageing and Retirement in Europe (SHARE) Wave 8. Release version: 8.0.0. SHARE-ERIC. Data set. doi: 10.6103/SHARE.w8.800.

## Ethics statement

The study was reviewed and approved by the Ethics Committee of the University of Mannheim and the Ethics Council of the Max Planck Society (http://www.share-project.org/fileadmin/pdf_documentation/SHARE_ethics_approvals.pdf). The patients/participants provided their written informed consent to participate in this study.

## Author contributions

DW-B developed the study concept, contributed to the data analysis, drafted, revised, and approved the final version of the manuscript. PB contributed to the data analysis, drafted, revised, and approved the final version of the manuscript. PS contributed to the study concept, drafted, revised, and approved the final version of the manuscript. All authors contributed to the article and approved the submitted version.
