# Supplementary material for: Mind-stimulating leisure activities: Prospective associations with health, wellbeing, and longevity
Source: Front Public Health. 2023 Feb 17;11:1117822. doi: 10.3389/fpubh.2023.1117822 (PMC9982162; doi:10.3389/fpubh.2023.1117822)
Supplement: Supplementary file 1 [file Table_1.DOCX]

Supplementary Material

Mind-Stimulating Leisure Activities: Prospective Associations with Health, Well-being, and Longevity

Dorota Weziak-Bialowolska^*^, Piotr Bialowolski, Pier Luigi Sacco

*** Correspondence:** Dorota Weziak-Bialowolska [doweziak@iq.harvard.edu](mailto:doweziak@iq.harvard.edu)

# Supplementary Information on Data

Longitudinal data from the Survey of Health, Ageing and Retirement in Europe (SHARE) (1) were used. SHARE is the largest social science panel conducted every two years to collect data on health, socioeconomic living conditions, and social and family networks of people aged 50 and over. The SHARE website (<http://www.share-project.org/data-documentation.html>) provides the study documentation and access to the data sets. The study was reviewed and approved by the Ethics Committee of the University of Mannheim and the Ethics Council of the Max Planck Society (http://www.share-project.org/fileadmin/pdf_documentation/ SHARE_ethics_approvals.pdf).

1. **Supplementary Information on** **Measures**

﻿**Leisure activities.** First, the relaxed leisure activity of reading was assessed by asking respondents about the frequency of reading books, magazines, or newspapers (almost every day, almost every week, almost every month, less often, never). For the analyses, this variable was recoded into three categories by keeping two extreme categories and merging the middle three (so that the resulting categories are almost every day, sometimes and never). Second, a serious leisure activity of playing word or number games, such as crossword puzzles or Sudoku, was also examined. Respondents provided information on their frequency of activity (almost every day, almost every week, almost every month, less often, no). As above, the three middle categories were merged and consequently, the three resulting categories to be used are: almost every day, sometimes, and never. Third, the social leisure activity of playing cards or playing games such as chess was considered. Once again, responses concerning the frequency were again grouped as almost every day, sometimes, and never (from the merging of the three middle categories featured in the original questionnaire).

**well-being outcomes.﻿** A sense of loneliness reflecting social isolation was measured using a 3-item loneliness scale, which is a short version of the UCLA Loneliness Scale (2). An example of a question is ’*How much of the time do you feel like you lack companionship?'.* Respondents provided information about the frequency of experiencing the feeling (1=hardly ever or never, 2=some of the time, and 3=often). The scale ranges from 3 to 9, with higher scores indicating higher levels of loneliness. Alternatively, loneliness was also measured using a single direct question about feeling lonely: ‘*How much of the time do you feel lonely?*’ (1=hardly ever or never, 2=some of the time, and 3=often).

A self-reported diagnosis of Alzheimer’s disease, dementia, or other serious memory impairment was also considered, as well as self-reported depression. To this end, the EURO-D geriatric depression scale (3–5) was used. This scale sums incidents of 12 depressive symptoms, including depression, pessimism, suicidality, guilt, sleep, interest, irritability, appetite, fatigue, concentration (on reading or entertainment), enjoyment, and tearfulness, and it ranges from 0 to 12. A score of 4 or higher is indicative of depression (4).

Additionally, five well-being related items of the CASP-12 questionnaire, which measures quality of life in early old age (4), were considered separately. We focused on the assessment of eudaimonic well-being reflected in sense of meaning (*‘I feel that my life has meaning*’), hedonic well-being reflected in happiness and feeling energetic (*‘I feel full of energy these days’;* ‘*On balance, I look back on my life with a sense of happiness*’) and optimism operationalized as positive expectations regarding the future (*‘Future looks good’* and *‘I look forward to each day’*). Respondents provided responses using a 4-point scale (often, sometimes, rarely, never).

**Daily life functioning.** Two daily life functioning indicators that measure the level of difficulty with activities of daily living (ADL) and instrumental activities of daily living (IADL) due to physical, mental, emotional, and memory problems were used. The ADL consist of six daily self-care activities that are crucial for maintaining independence such as dressing, walking, grooming, eating, transferring to bed, and toileting. IADL consist of seven more complex activities such as preparing hot meals, shopping for groceries, making telephone calls, taking medications, carrying out work around the house and garden, using a map to navigate in an unknown location, and managing one’s money (e.g., paying bills, keeping track of expenses) (4,6). The scores range from 0 to 6 and 7, respectively. Values above 0 indicate reported limitations with ADL and/or IADL.

**Physical health outcomes**. The following physical health outcomes were examined: heart attack, hypertension, high blood cholesterol, stroke, diabetes, and cancer. These variables accounted for both nonfatal and fatal medical conditions and were constructed using (i) self-reports of the presence or absence of a doctor diagnosis for these medical conditions from the main survey and (ii) exit interviews reporting the cause of death.

Furthermore, we considered two additional variables: the presence of impairing pain (‘*Do you feel troubled by pain?*’; yes or no), and whether the respondents experienced at least one mobility, arm or fine motor limitation (yes or no).

**Cognitive impairment.** Cognitive impairment was considered using a measure of time orientation. Specifically, respondents were asked a series of questions about awareness of current year, month, and day of the month, as well as about day of the week. This summary measure ranged from 0=bad to 4=good (7).

**All-cause mortality.** Mortality, regardless of the cause of death, was also considered an outcome. Information on the incidence of death was derived from exit interviews and was monitored starting from the baseline wave (wave 5) up to wave 8.

**Covariates.** All covariates were self-reported and measured in the pre-baseline wave (i.e., wave 4). They included demographic and socioeconomic factors, personality traits, health behaviors, lifestyle factors, and previous health history, as these variables were found in previous research as possible confounders of the associations examined. Demographic factors included gender (male and female), age (50–59, 60–69, 70–79, 80+), marital status (married and living with spouse, registered partnership, married but living separate from spouse, never married, divorced, widowed), highest educational attainment (according to the International Standard Classification of Education, version 1997 [ISCED-97], as available in the SHARE datasets), and country. Socioeconomic variables included annual personal income (after logarithmic transformation to account for skewness and outliers) and net financial assets of the household (after logarithmic transformation to account for skewness and outliers), The personality traits included agreeableness, openness, neuroticism, conscientiousness, and extraversion measured using the Big Five Inventory (BFI-10) (8). The health behaviors accounted for included: sports activity requiring a moderate level of effort (more than once a week, once a week, one to three times a month, hardly ever or never), alcohol consumption (almost every day, five or six days a week, three or four days a week, once or twice a week, once or twice a month, less than once a month, not at all in the last 6 months) and BMI. Lifestyle factors included volunteering (‘*Have you done voluntary or charity work in the last 12 months?*’; yes or no).

﻿**Prior values of outcomes and exposure.** To reduce the possibility of reverse causation and residual confounding, we adjusted for the prior values of the 21 outcome variables (i.e., prior emotional well-being, quality of life, cognitive impairment, and history of the diseases examined). Additionally, we also controlled for prior values of the respective exposure variable

# Supplementary Tables

**Table S1**. Distribution of Participant Characteristics at Study Prebaseline Wave by Leisure Activity (wave 4, N=19,821). Survey of Health, Ageing and Retirement in Europe (SHARE), Middle-Aged and Older Adults Aged 50 and More.

| **Participant Characteristic** | **Reading books, magazines and newspapers** | | | | **Doing number and word games** | | | | **Playing cards and games** | | | |
| --- | --- | --- | --- | --- | --- | --- | --- | --- | --- | --- | --- | --- |
|  | % | Mean (SD) | % | Mean (SD) | % | Mean (SD) | % | Mean (SD) | % | Mean (SD) | % | Mean (SD) |
|  | No (N=4,186) | | Yes (N=15,516) | | No (N=10,420) | | Yes (N=9,283) | | No (N=13,513) | | Yes (N=6,190) | |
| ***Sociodemographic factors*** |  |  |  |  |  |  |  |  |  |  |  |  |
| Gender |  |  |  |  |  |  |  |  |  |  |  |  |
| Male | 45.7 |  | 39.3 |  | 47.2 |  | 33.4 |  | 39.9 |  | 42.3 |  |
| Female | 54.3 |  | 60.7 |  | 52.8 |  | 66.6 |  | 60.1 |  | 57.7 |  |
| Age group |  |  |  |  |  |  |  |  |  |  |  |  |
| 50-59 | 30.7 |  | 31.2 |  | 31.6 |  | 30.6 |  | 29.7 |  | 34.0 |  |
| 60-69 | 37.6 |  | 40.8 |  | 38.1 |  | 42.3 |  | 39.6 |  | 41.1 |  |
| 70-79 | 25.0 |  | 22.9 |  | 24.1 |  | 22.5 |  | 24.6 |  | 20.6 |  |
| 80+ | 6.7 |  | 5.2 |  | 6.2 |  | 4.7 |  | 6.1 |  | 4.2 |  |
| Marital status |  |  |  |  |  |  |  |  |  |  |  |  |
| married and living together with spouse | 72.2 |  | 69.0 |  | 70.4 |  | 68.9 |  | 68.9 |  | 71.5 |  |
| registered partnership | 1.5 |  | 1.5 |  | 1.4 |  | 1.6 |  | 1.4 |  | 1.7 |  |
| married but living separate | 1.3 |  | 1.3 |  | 1.4 |  | 1.3 |  | 1.3 |  | 1.3 |  |
| never married | 5.9 |  | 5.4 |  | 6.0 |  | 5.0 |  | 5.8 |  | 4.9 |  |
| divorced | 6.8 |  | 10.4 |  | 8.7 |  | 10.8 |  | 9.2 |  | 10.6 |  |
| widowed | 12.3 |  | 12.4 |  | 12.2 |  | 12.6 |  | 13.5 |  | 10.0 |  |
| Education attainment (ISCED-97) |  |  |  |  |  |  |  |  |  |  |  |  |
| None | 7.1 |  | 0.9 |  | 3.6 |  | 0.7 |  | 2.7 |  | 1.3 |  |
| Primary education or first stage of basic education | 30.7 |  | 10.8 |  | 20.0 |  | 9.5 |  | 16.4 |  | 11.9 |  |
| Lower secondary or second stage of basic education | 20.2 |  | 17.3 |  | 19.5 |  | 16.2 |  | 19.4 |  | 14.9 |  |
| (Upper) secondary education | 30.2 |  | 37.8 |  | 32.8 |  | 40.0 |  | 35.0 |  | 38.8 |  |
| Post-secondary non-tertiary education | 2.8 |  | 6.3 |  | 4.8 |  | 6.4 |  | 5.4 |  | 5.9 |  |
| First stage of tertiary education | 8.4 |  | 25.9 |  | 18.5 |  | 26.4 |  | 20.4 |  | 26.2 |  |
| Second stage of tertiary education | 0.5 |  | 0.9 |  | 0.8 |  | 0.8 |  | 0.8 |  | 1.0 |  |
| Annual personal income (Euro) |  | 22,589 (38,255) |  | 34,640 (50,135) |  | 30,322 (47,895) |  | 34,045 (48,276) |  | 28,316 (45,491) |  | 40,291 (52,457) |
| Household net financial assets (Euro) |  | 28,447 (117,926) |  | 69,420 (206,897) |  | 53,706 (193,145) |  | 68,570 (190,860) |  | 50,073 (177,100) |  | 83,937 (219,877) |
| Country |  |  |  |  |  |  |  |  |  |  |  |  |
| Austria | 4.1 |  | 8.0 |  | 6.7 |  | 7.7 |  | 5.7 |  | 10.2 |  |
| Germany | 2.0 |  | 3.6 |  | 3.1 |  | 3.5 |  | 2.8 |  | 4.4 |  |
| Sweden | 1.0 |  | 5.2 |  | 3.0 |  | 5.9 |  | 3.9 |  | 5.3 |  |
| The Netherlands | 2.4 |  | 6.1 |  | 4.0 |  | 6.8 |  | 4.4 |  | 7.2 |  |
| Spain | 14.6 |  | 3.7 |  | 9.4 |  | 2.3 |  | 6.7 |  | 4.6 |  |
| Italy | 16.8 |  | 2.8 |  | 8.9 |  | 2.2 |  | 6.8 |  | 3.4 |  |
| France | 14.4 |  | 9.7 |  | 11.0 |  | 10.5 |  | 10.5 |  | 11.2 |  |
| Denmark | 2.0 |  | 6.2 |  | 3.9 |  | 6.9 |  | 3.8 |  | 8.7 |  |
| Switzerland | 4.0 |  | 10.5 |  | 8.3 |  | 10.0 |  | 7.2 |  | 13.2 |  |
| Belgium | 4.7 |  | 7.4 |  | 6.7 |  | 7.0 |  | 6.5 |  | 7.6 |  |
| Czech Republic | 7.5 |  | 10.8 |  | 7.5 |  | 13.1 |  | 10.7 |  | 8.9 |  |
| Poland | 6.2 |  | 2.3 |  | 4.3 |  | 1.9 |  | 4.1 |  | 1.0 |  |
| Hungary | 4.0 |  | 3.7 |  | 4.2 |  | 3.2 |  | 4.2 |  | 2.8 |  |
| Slovenia | 10.9 |  | 4.5 |  | 7.2 |  | 4.4 |  | 7.3 |  | 2.7 |  |
| Estonia | 5.4 |  | 15.6 |  | 12.2 |  | 14.8 |  | 15.5 |  | 8.8 |  |
| ***Personality traits*** |  |  |  |  |  |  |  |  |  |  |  |  |
| Extraversion; 1-5 |  | 3.4 (0.9) |  | 3.5 (0.9) |  | 3.5 (0.9) |  | 3.5 (0.9) |  | 3.5 (0.9) |  | 3.6 (0.9) |
| Agreeableness; 1-5 |  | 3.7 (0.8) |  | 3.7 (0.8) |  | 3.7 (0.8) |  | 3.7 (0.8) |  | 3.7 (0.8) |  | 3.7 (0.8) |
| Consciousness; 1-5 |  | 4.1 (0.8) |  | 4.1 (0.8) |  | 4.1 (0.8) |  | 4.1 (0.8) |  | 4.1 (0.8) |  | 4.1 (0.8) |
| Neuroticism; 1-5 |  | 2.7 (1.0) |  | 2.5 (1.0) |  | 2.6 (1.0) |  | 2.5 (1.0) |  | 2.6 (1.0) |  | 2.5 (1.0) |
| Openness; 1-5 |  | 3.1 (0.9) |  | 3.4 (1.0) |  | 3.3 (1.0) |  | 3.5 (1.0) |  | 3.3 (1.0) |  | 3.4 (1.0) |
| ***Lifestyle factors*** |  |  |  |  |  |  |  |  |  |  |  |  |
| BMI |  | 27.6 (5.0) |  | 26.8 (5.4) |  | 27.1 (4.7) |  | 26.9 (4.8) |  | 27.1 (4.8) |  | 26.9 (4.7) |
| Volunteering or charity work (yes) | 10.4 |  | 21.8 |  | 16.2 |  | 23.0 |  | 16.2 |  | 26.5 |  |
| Alcohol consumption |  |  |  |  |  |  |  |  |  |  |  |  |
| Almost every day | 18.8 |  | 17.1 |  | 19.2 |  | 15.5 |  | 16.9 |  | 18.7 |  |
| 5-6 days a week | 2.9 |  | 3.0 |  | 3.0 |  | 3.0 |  | 2.7 |  | 3.6 |  |
| 3-4 days a week | 5.1 |  | 8.1 |  | 6.6 |  | 8.4 |  | 6.4 |  | 9.8 |  |
| once or twice a week | 12.9 |  | 21.4 |  | 17.3 |  | 22.2 |  | 16.9 |  | 25.4 |  |
| once or twice a month | 8.1 |  | 14.5 |  | 11.0 |  | 15.6 |  | 12.3 |  | 15.0 |  |
| less than once a month | 9.5 |  | 12.3 |  | 10.7 |  | 12.9 |  | 12.1 |  | 10.9 |  |
| not at all in the last 6 months | 42.8 |  | 23.6 |  | 32.4 |  | 22.4 |  | 32.8 |  | 16.6 |  |
| Sport activity requiring a moderate level of energy |  |  |  |  |  |  |  |  |  |  |  |  |
| more than once a week | 59.8 |  | 76.9 |  | 69.3 |  | 77.8 |  | 70.1 |  | 79.0 |  |
| once a week | 15.1 |  | 12.9 |  | 14.1 |  | 12.6 |  | 13.8 |  | 12.4 |  |
| one to three times a month | 8.1 |  | 4.7 |  | 6.1 |  | 4.6 |  | 5.9 |  | 4.3 |  |
| hardly ever or never | 17.0 |  | 5.5 |  | 10.5 |  | 5.0 |  | 9.6 |  | 4.4 |  |
| **Outcomes** |  |  |  |  |  |  |  |  |  |  |  |  |
| Well-being |  |  |  |  |  |  |  |  |  |  |  |  |
| Loneliness (3-item loneliness scale) |  | 3.9 (1.5) |  | 3.7 (1.1) |  | 3.8 (1.3) |  | 3.6 (1.1) |  | 3.8 (1.3) |  | 3.6 (1.0) |
| Alzheimer’s disease | 1.1 |  | 0.3 |  | 0.6 |  | 0.3 |  | 0.6 |  | 0.2 |  |
| Depression (EURO-D≥4) | 32.9 |  | 23.2 |  | 27.5 |  | 22.9 |  | 27.5 |  | 20.6 |  |
| Future looks good (1-4) |  | 2.9 (0.9) |  | 3.2 (0.9) |  | 3.1 (0.9) |  | 3.2 (0.9) |  | 3.0 (0.9) |  | 3.3 (0.8) |
| I feel full of energy these days (1-4) |  | 3.1 (0.9) |  | 3.3 (0.8) |  | 3.2 (0.8) |  | 3.3 (0.8) |  | 3.2 (0.8) |  | 3.4 (0.7) |
| On balance, I look back on my life with a sense of happiness (1-4) |  | 3.3 (0.8) |  | 3.4 (0.7) |  | 3.4 (0.8) |  | 3.4 (0.7) |  | 3.4 (0.8) |  | 3.5 (0.7) |
| I look forward to each day (1-4) |  | 3.2 (0.9) |  | 3.6 (0.8) |  | 3.4 (0.9) |  | 3.6 (0.8) |  | 3.4 (0.9) |  | 3.6 (0.7) |
| I feel that my life has meaning (1-4) |  | 3.4 (0.8) |  | 3.7 (0.6) |  | 3.6 (0.7) |  | 3.7 (0.6) |  | 3.6 (0.7) |  | 3.7 (0.6) |
| Daily life functioning |  |  |  |  |  |  |  |  |  |  |  |  |
| ADL  (at least 1 limitation) | 18.4 |  | 10.5 |  | 8.3 |  | 6.7 |  | 8.2 |  | 6.1 |  |
| IADL  (at least 1 limitation) | 9.6 |  | 7.0 |  | 13.8 |  | 10.4 |  | 13.4 |  | 9.5 |  |
| Physical health |  |  |  |  |  |  |  |  |  |  |  |  |
| Heart attack | 11.8 |  | 10.7 |  | 11.6 |  | 10.2 |  | 11.9 |  | 9.0 |  |
| Hypertension | 40.2 |  | 38.1 |  | 38.4 |  | 38.6 |  | 39.7 |  | 35.9 |  |
| High blood cholesterol | 23.2 |  | 22.3 |  | 21.9 |  | 23.2 |  | 22.4 |  | 22.8 |  |
| Stroke | 3.3 |  | 2.6 |  | 3.1 |  | 2.5 |  | 3.0 |  | 2.3 |  |
| Diabetes | 13.2 |  | 9.9 |  | 11.4 |  | 9.7 |  | 11.3 |  | 9.1 |  |
| Cancer | 2.7 |  | 4.5 |  | 3.5 |  | 4.8 |  | 3.9 |  | 4.7 |  |
| Pain | 53.0 |  | 39.9 |  | 44.7 |  | 40.3 |  | 44.7 |  | 38.2 |  |
| Mobility (at least 1 limitation) | 51.4 |  | 44.0 |  | 46.3 |  | 44.8 |  | 47.1 |  | 42.3 |  |
| Cognitive Impairment |  |  |  |  |  |  |  |  |  |  |  |  |
| Time Orientation |  | 3.8 (0.6) |  | 3.9 (0.4) |  | 3.8 (0.5) |  | 3.9 (0.4) |  | 3.8 (0.5) |  | 3.9 (0.4) |

SD=standard deviation, BMI-body mass index; No=Never, Yes=Sometimes or Almost every day

**Table S2**. Prospective Associations Between Reading Books, Magazines or Newspapers, Doing Word and Number Games and Playing Cards or Games and Well-Being, Physical Health, Daily Life Functioning, Cognitive Impairment and All-cause Mortality - After Excluding Respondents with a Pre-Baseline Health Condition. Survey of Health, Ageing and Retirement in Europe (SHARE), Adults aged 50 and Over.

|  |  | **Reading books, magazines or newspapers**  **(ref.= Never)** | | **Doing word or number games (ref.= Never)** | | **Playing cards or games**  **(ref.= Never)** | |
| --- | --- | --- | --- | --- | --- | --- | --- |
| **Outcome** | **Statistics** | **Sometimes** | **Almost every day** | **Sometimes** | **Almost every day** | **Sometimes** | **Almost every day** |
| Emotional well-being |  |  |  |  |  |  |  |
| Alzheimer’s disease  (N=19,917) | OR  (95% CI)  p-value | 0.844  (0.584, 1.219)  0.363 | 0.674  (0.42, 1.025)  0.065 | 0.810  (0.599, 1.095)  0.168 | 0.840  (0.623, 1.133)  0.253 | 0.816  (0.628, 1.094)  0.155 | 0.579^†^  (0.375, 0.894)  0.014 |
| Depression (EURO-D≥4) (N=14,753) | RR  (95% CI)  p-value | 0.958  (0.830, 1.105)  0.553 | 0.914  (0.821, 1.017)  0.099 | 0.986^†^  (0.819, 0.981)  0.018 | 0.918  (0.839, 1.006)  0.066 | 0.967^†^  (0.783, 0.961)  0.007 | 0.896  (0.757, 1.060)  0.200 |
| Physical health |  |  |  |  |  |  |  |
| Heart attack (N=17,644) | OR  (95% CI)  p-value | 0.950  (0.825, 1.096)  0.482 | 1.036  (0.899, 1.194)  0.619 | 0.987  (0.865, 1.125)  0.840 | 1.076  (0.955, 1.211)  0.226 | 0.988  (0.890; 1.097)  0.821 | 0.894  (0.711, 1.124)  0.338 |
| Hypertension (N=12,191) | RR  (95% CI)  p-value | 0.928  (0.813, 1.060)  0.273 | 0.955  (0.839, 1.088)  0.490 | 1.033  (0.952, 1.020)  0.400 | 0.967  (0.866, 1.081)  0.556 | 0.995  (0.916, 1.081)  0.905 | 0.991  (0.816, 1.204)  0.929 |
| High blood cholesterol (N=15,351) | RR  (95% CI)  p-value | 1.002  (0.853, 1.177)  0.979 | 0.908  (0.810, 1.018)  0.098 | 1.024  (0.926, 1.132)  0.641 | 1.029  (0.917, 1.154)  0.624 | 1.008  (0.926, 1.097)  0.860 | 0.948  (0.792, 1.135)  0.560 |
| Stroke (N=19,262) | OR  (95% CI)  p-value | 0.881  (0.612, 1.270)  0.496 | 0.883  (0.666, 1.172)  0.387 | 0.979  (0.757, 1.267)  0.873 | 0.908  (0.669, 1.233)  0.536 | 1.236  (0.989, 1.544)  0.062 | 1.326  (0.927, 1.896)  0.122 |
| Diabetes (N=17.722) | RR  (95% CI)  p-value | 0.931  (0.770, 1.126)  0.462 | 1.073  (0.907, 1.270)  0.407 | 0.992  (0.897, 1.060)  0.870 | 1.058  (0.904, 1.238)  0.484 | 0.927  (0.785, 1.095)  0.371 | 0.819  (0.637, 1.052)  0.117 |
| Cancer (n=19,005) | RR  (95% CI)  p-value | 0.803  (0.621, 1.039)  0.094 | 1.076  (0.897, 1.289)  0.430 | 0.901  (0.704, 1.153)  0.405 | 0.979  (0.787, 1.217)  0.847 | 1.198^†^  (0.993, 1.445)  0.059 | 1.458^†^  (1.072, 1.983)  0.016 |
| All-cause mortality | OR  (95% CI)  p-value | 1.022  (0.761, 1.373)  0.884 | 0.852  (0.628, 1.156)  0.303 | 0.817  (0.613, 1.090)  0.169 | 0.740  (0.529, 1.036)  0.080 | 0.934  (0.712, 1. 226)  0.624 | 0.981  (0.656, 1.468)  0.927 |

CI, confidence interval; OR, odds ratio; RR, risk ratio; ADL, activities of daily living; IADL, instrumental activities of daily living.

^a^ Missing covariate variables were imputed using chained equations (ten sets of imputed data were generated). All models were controlled for participant demographics: age, gender, marital status, educational attainment, and country; socioeconomic factors: annual personal income, household net financial assets, health behaviors such as BMI, alcohol consumption, and sports activity; lifestyle factors demonstrated in volunteer activities; and personality traits such as agreeableness, openness, conscientiousness, neuroticism, and extraversion. Each model was also adjusted for prior values of the 21 outcome variables and of the exposure variable, as well as previous self-reported presence/absence of diagnosis for heart attack, hypertension, high blood cholesterol, stroke, diabetes, and cancer simultaneously in each regression model.

^b^ All continuous outcomes were standardized (mean = 0, standard deviation = 1), and β was the standardized effect size.

^c^ p<0.05 after Bonferroni correction (p-value cut-off for Bonferroni correction = 0.05/21 outcomes=0.0024)

^†^ Not significant after Bonferroni correction.

**Table S3**. Prospective Associations Between Reading Books, Magazines or Newspapers, Doing Word and Number Games and Playing Cards or Games and Emotional Well-Being, Physical Health, Daily Life Functioning, Cognitive Impairment and All-cause Mortality – Complete Case Scenario. Survey of Health, Ageing and Retirement in Europe (SHARE), Adults aged 50 and Over (N=8,134-8,343).

|  |  | **Reading books, magazines or newspapers**  **(ref.= Never)** | | **Doing word or number games (ref.= Never)** | | **Playing cards or games**  **(ref.= Never)** | |
| --- | --- | --- | --- | --- | --- | --- | --- |
| **Outcome** | **Statistics** | **Sometimes** | **Almost every day** | **Sometimes** | **Almost every day** | **Sometimes** | **Almost every day** |
| Emotional well-being |  |  |  |  |  |  |  |
| Loneliness (3-item loneliness scale) | $\beta^{b}$  (95% CI)  p-value | -0.008  (-0.106, 0.090)  0.867 | -0.052  (-0.118, 0.015)  0.113 | -0.031  (-0.075, 0.013)  0.149 | -0.038  (-0.100, 0.024)  0.208 | -0.073^†^  (-0.115, -0.032)  0.003 | -0.033  (-0.141, 0.076)  0.520 |
| Alzheimer’s disease | OR  (95% CI)  p-value | 1.370  (0.888, 2.113)  0.154 | 1.375  (0.890, 2.124)  0.151 | 0.891  (0.665, 1.195)  0.441 | 0.958  (0.782, 1.173)  0.675 | 0.642^†^  (0.426, 0.967)  0.034 | 0.654  (0.359, 1.192)  0.165 |
| Depression (EURO-D≥4) | RR  (95% CI)  p-value | 0.969  (0.922, 1.019)  0.221 | 0.895  (0.836, 0.959)  0.002 | 0.891^†^  (0.799, 0.994)  0.038 | 0.831  (0.771, 0.897)  <0.001 | 0.880  (0.825, 0.937)  <0.001 | 0.823  (0.737, 0.919)  0.001 |
| Future looks good | $\beta^{b}$  (95% CI)  p-value | 0.059  (-0.016, 0.133)  0.008 | 0.098^†^  (0.013, 0.183)  0.028 | 0.054^†^  (0.010, 0.098)  0.021 | 0.067  (-0.012, 0.147)  0.088 | 0.041  (-0.031, 0.113)  0.236 | 0.061  (-0.065, 0.187)  0.310 |
| I feel full of energy these days | $\beta^{b}$  (95% CI)  p-value | 0.038  (-0.007, 0.082)  0.092 | 0.098^†^  (0.036, 0.161)  0.005 | 0.024  (-0.013, 0.060)  0.179 | 0.059^†^  (0.005, 0.112)  0.035 | 0.025  (-0.030, 0.081)  0.331 | -0.024  (-0.149, 0.102)  0.687 |
| On balance, I look back on my life with a sense of happiness | $\beta^{b}$  (95% CI)  p-value | 0.097  (-0.010, 0.207)  0.071 | 0.138  (0.090, 0.185)  <0.001 | -0.011  (-0.043, 0.021)  0.474 | 0.026  (-0.071, 0.124)  0.564 | 0.052  (-0.022, 0.126)  0.152 | 0.130^†^  (0.017, 0.242)  0.028 |
| I look forward to each day | $\beta^{b}$  (95% CI)  p-value | 0.053^†^  (0.001, 0.104)  0.045 | 0.087^†^  (0.025, 0.150)  0.010 | 0.050^†^  (0.013, 0.086)  0.013 | 0.070^†^  (0.004, 0.135)  0.039 | 0.081^†^  (0.030, 0.132)  0.005 | -0.019  (-0.114, 0.076)  0.671 |
| I feel that my life has meaning | $\beta^{b}$  (95% CI)  p-value | 0.042  (-0.036, 0.120)  0.262 | 0.103  (0.048, 0.157)  0.002 | -0.005  (-0.040, 0.030)  0.768 | 0.025  (-0.037, 0.086)  0.396 | 0.028  (-0.058, 0.115)  0.487 | -0.043  (-0.195, 0.108)  0.543 |
| Daily life functioning |  |  |  |  |  |  |  |
| ADL  (at least 1 limitation) | RR  (95% CI)  p-value | 0.956  (0.831, 1.102)  0.546 | 0.973^†^  (0.832, 1.139)  0.738 | 0.916  (0.772, 1.088)  0.318 | 0.960  (0.752, 1.224)  0.741 | 0.984  (0.830, 1.166)  0.849 | 0.981  (0.803, 1.198)  0.852 |
| IADL  (at least 1 limitation) | RR  (95% CI)  p-value | 0.862^†^  (0.757, 0.982)  0.026 | 0.907  (0.775, 1.061)  0.223 | 0.988  (0.899, 1.087)  0.813 | 0.878  (0.728, 1.058)  0.171 | 0.897  (0.769, 1.046)  0.164 | 0.942  (0.762, 1.165)  0.582 |
| Physical health |  |  |  |  |  |  |  |
| Heart attack | OR  (95% CI)  p-value | 0.911  (0.837, 0.991)  0.030 | 0.977  (0.873, 1.094)  0.690 | 0.983  (0.872, 1.109)  0.781 | 1.094  (0.957, 1.249)  0.187 | 1.048  (0.960; 1.144)  0.292 | 1.128  (0.844, 1.507)  0.416 |
| Hypertension | RR  (95% CI)  p-value | 0.969  (0.875, 1.073)  0.549 | 0.971(0.905, 1.042)  0.417 | 0.995  (0.965, 1.026)  0.732 | 0.984  (0.937, 1.033)  0.514 | 1.026  (0.967, 1.090)  0.396 | 1.067  (0.910, 1.252)  0.424 |
| High blood cholesterol | RR  (95% CI)  p-value | 0.968  (0.819, 1.143)  0.701 | 0.920  (0.834, 1.015)  0.097 | 1.045  (0.951, 1.148)  0.358 | 1.038  (0.923, 1.168)  0.534 | 1.067^†^  (1.004, 1.136)  0.038 | 0.960  (0.851, 1.082)  0.502 |
| Stroke | OR  (95% CI)  p-value | 0.852  (0.530, 1.368)  0.506 | 0.824  (0.621, 1.092)  0.177 | 0.906  (0.757, 1.085)  0.285 | 0.899  (0.757, 1.142)  0.651 | 1.161  (0.920, 1.466)  0.208 | 1.561  (0.795, 3.065)  0.196 |
| Diabetes | RR  (95% CI)  p-value | 0.987  (0.878, 1.110)  0.834 | 1.165^†^  (1.047, 1.295)  0.005 | 1.002  (0.928, 1.082)  0.959 | 1.047  (0.895, 1.224)  0.568 | 1.040  (0.966, 1.120)  0.297 | 1.050  (0.868, 1.270)  0.616 |
| Cancer | RR  (95% CI)  p-value | 0.845  (0.575, 1.243)  0.394 | 1.082  (0.797, 1.470)  0.612 | 0.983  (0.872, 1.109)  0.781 | 1.094  (0.957, 1.249)  0.187 | 1.104  (0.917, 1.329)  0.298 | 1.571  (0.982, 2.513)  0.059 |
| Pain | RR  (95% CI)  p-value | 0.995  (0.924, 1.073)  0.908 | 0.946  (0.890, 1.006)  0.079 | 0.970  (0.934, 1.008)  0.124 | 0.911^†^  (0.845, 0.983)  0.016 | 0.932^†^  (0.890, 0.976)  0.003 | 1.007  (0.895, 1.133)  0.911 |
| Mobility | RR  (95% CI)  p-value | 1.016  (0.965, 1.071)  0.539 | 0.985  (0.941, 1.031)  0.523 | 0.999  (0.966, 1.032)  0.951 | 0.938  (0.919, 0.957)  <0.001 | 0.937^†^  (0.901, 0.980)  0.004 | 1.008  (0.926, 1.096)  0.861 |
| Cognitive Impairment | $\beta^{b}$  (95% CI)  p-value | 0.020  (-0.049, 0.088)  0.540 | 0.016  (-0.034, 0.065)  0.502 | 0.057^†^  (0.023, 0.090)  0.003 | 0.024  (-0.024, 0.072)  0.293 | 0.033  (-0.004, 0.071)  0.073 | 0.068  (-0.021, 0.157)  0.121 |
| All-cause mortality | OR  (95% CI)  p-value | 0.917  (0.667, 1.261)  0.595 | 0.870  (0.736, 1.028)  0.102 | 0.858  (0.688, 1.070)  0.174 | 0.940  (0.804, 1.099)  0.440 | 0.940  (0.708, 1.247)  0.668 | 0.880  (0.633, 1.225)  0.449 |

CI, confidence interval; OR, odds ratio; RR, risk ratio; ADL, activities of daily living; IADL, instrumental activities of daily living.

^a^ All models were controlled for participant demographics: age, gender, marital status, educational attainment, and country; socioeconomic factors: annual personal income, household net financial assets, health behaviors such as BMI, alcohol consumption, and sports activity; lifestyle factors demonstrated in volunteer activities; and personality traits such as agreeableness, openness, conscientiousness, neuroticism, and extraversion. Each model was also adjusted for prior values of the 21 outcome variables and of the exposure variable, as well as previous self-reported presence/absence of diagnosis for heart attack, hypertension, high blood cholesterol, stroke, diabetes, and cancer simultaneously in each regression model.

^b^ All continuous outcomes were standardized (mean = 0, standard deviation = 1), and β was the standardized effect size.

^c^ p<0.05 after Bonferroni correction (p-value cut-off for Bonferroni correction = 0.05/21 outcomes=0.0024)

^†^ Not significant after Bonferroni correction.

**Table S4**. Prospective Associations Between Reading Books, Magazines or Newspapers, Doing Word and Number Games and Playing Cards or Games and Emotional Well-Being, Physical Health, Daily Life Functioning, Cognitive Impairment and All-cause Mortality – Limited Set of Controls. Survey of Health, Ageing and Retirement in Europe (SHARE), Adults aged 50 and Over (N=19,821).

|  |  | **Reading books, magazines or newspapers**  **(ref.= Never)** | | **Doing word or number games (ref.= Never)** | | **Playing cards or games**  **(ref.= Never)** | |
| --- | --- | --- | --- | --- | --- | --- | --- |
| **Outcome** | **Statistics** | **Sometimes** | **Almost every day** | **Sometimes** | **Almost every day** | **Sometimes** | **Almost every day** |
| Emotional well-being |  |  |  |  |  |  |  |
| Loneliness (3-item loneliness scale) | $\beta^{b}$  (95% CI)  p-value | -0.051  (-0.111, 0.008)  0.084 | -0.132  (-0.190, -0.075)  <0.001 | -0.047  (-0.100, 0.006)  0.075 | -0.052  (-0.109, 0.004)  0.066 | -0.100  (-0.134, -0.066)  <0.001 | -0.132  (-0.182, -0.081)  <0.001 |
| Alzheimer’s disease | OR  (95% CI)  p-value | 0.827  (0.588, 1.263)  0.273 | 0.601^†^  (0.414, 0.873)  0.008 | 0.751  (0.559, 1.009)  0.057 | 0.756  (0.561, 1.018)  0.065 | 0.771  (0.591, 1.006)  0.055 | 0.606^†^  (0.427, 0.861)  0.005 |
| Depression (EURO-D≥4) | RR  (95% CI)  p-value | 0.938  (0.879, 1.002)  0.056 | 0.828  (0.770, 0.891)  <0.001 | 0.886^†^  (0.831, 0.944)  0.003 | 0.890  (0.825, 0.960)  <0.001 | 0.860  (0.799, 0.926)  <0.001 | 0.924  (0.844, 1.011)  0.087 |
| Future looks good | $\beta^{b}$  (95% CI)  p-value | 0.092^†^  (0.037, 0.147)  0.004 | 0.183  (0.123, 0.242)  <0.001 | 0.073^†^  (0.020, 0.125)  0.011 | 0.082^†^  (0.016, 0.148)  0.020 | 0.082  (0.044, 0.121)  0.001 | 0.036  (-0.025, 0.097)  0.216 |
| I feel full of energy these days | $\beta^{b}$  (95% CI)  p-value | 0.057^†^  (0.002, 0.112)  0.045 | 0.174  (0.108, 0.240)  <0.001 | 0.071^†^  (0.028, 0.114)  0.005 | 0.077^†^  (0.027, 0.128)  0.006 | 0.061^†^  (0.024, 0.098)  0.004 | -0.018  (-0.078, 0.041)  0.496 |
| On balance, I look back on my life with a sense of happiness | $\beta^{b}$  (95% CI)  p-value | 0.092^†^  (0.025, 0.158)  0.012 | 0.196  (0.144, 0.253  <0.001 | 0.036  (-0.001, 0.074)  0.057 | 0.044  (-0.021, 0.107)  0.163 | 0.076^†^  (0.030, 0.127)  0.005 | 0.106^†^  (0.042, 0.170)  0.004 |
| I look forward to each day | $\beta^{b}$  (95% CI)  p-value | 0.097^†^  (0.030, 0.163)  0.008 | 0.167  (0.111, 0.222)  <0.000 | 0.088  (0.050, 0.125)  <0.001 | 0.076^†^  (0.026, 0.127)  0.007 | 0.099  (0.065, 0.133)  <0.001 | 0.034  (-0.038, 0.106)  0.570 |
| I feel that my life has meaning | $\beta^{b}$  (95% CI)  p-value | 0.069^†^  (0.009, 0.129)  0.029 | 0.186  (0.124, 0.248)  <0.001 | 0.049^†^  (0.016, 0.082)  0.007 | 0.045^†^  (0.002, 0.087)  0.041 | 0.068^†^  (0.012, 0.123)  0.021 | -0.005  (-0.099, 0.099)  0.917 |
| Daily life functioning |  |  |  |  |  |  |  |
| ADL  (at least 1 limitation) | RR  (95% CI)  p-value | 0.894  (0.794, 1.007)  0.065 | 0.769  (0.688, 0.859)  <0.001 | 0.880^†^  (0.789, 0.982)  0.022 | 0.885^†^  (0.795, 0.985)  0.025 | 0.907^†^  (0.836, 0.984)  0.019 | 1.090  (0.925, 1.284)  0.302 |
| IADL  (at least 1 limitation) | RR  (95% CI)  p-value | 0.883^†^  (0.805, 0.968)  0.008 | 0.821  (0770, 0.875)  <0.001 | 0.889^†^  (0.806, 0.981)  0.020 | 0.880^†^  (0.789, 0.981)  0.022 | 0.960^†^  (0.835, 0.989)  0.027 | 1.034  (0.897, 1.192)  0.642 |
| Physical health |  |  |  |  |  |  |  |
| Heart attack | OR  (95% CI)  p-value | 0.943  (0.825, 1.076)  0.380 | 0.938  (0.832, 1.056)  0.289 | 0.919  (0.826, 1.022)  0.118 | 0.978  (0891, 1.073)  0.632 | 1.000  (0.909; 1.099)  0.995 | 0.993  (0.794, 1.243)  0.953 |
| Hypertension | RR  (95% CI)  p-value | 0.986  (0.908, 1.070)  0.734 | 0.958  (0.901, 1.019)  0.171 | 1.023  (0.970, 1.080)  0.423 | 1.019  (0.969, 1.073)  0.460 | 0.986  (0.947, 1.027)  0.499 | 1.021  (0.904, 1.153)  0.736 |
| High blood cholesterol | RR  (95% CI)  p-value | 0.992  (0.893, 1.101)  0.873 | 0.908  (0.822, 1.001)  0.053 | 0.981  (0.922, 1.042)  0.528 | 1.043  (0.974, 1.118)  0.228 | 1.010  (0.958, 1.065)  0.712 | 1.060  (0.956, 1.176)  0.267 |
| Stroke | OR  (95% CI)  p-value | 0.825  (0.624, 1.091)  0.177 | 0.703^†^  (0.548, 0.902)  0.006 | 0.865  (0.694, 1.077)  0.192 | 0.827  (0.654, 1.045)  0.111 | 1.097  (0.887, 1.358)  0.392 | 1.228  (0.855, 1.765)  0.266 |
| Diabetes | RR  (95% CI)  p-value | 0.891  (0.775, 1.025)  0.107 | 0.966  (0.852, 1.095)  0.587 | 0.965  (0.882, 1.055)  0.424 | 1.019  (0.907, 1.145)  0.784 | 0.936  (0.856, 1.024)  0.147 | 1.120  (0.979, 1.281)  0.098 |
| Cancer | RR  (95% CI)  p-value | 0.825  (0.644, 1.056)  0.124 | 1.009  (0.828, 1.231)  0.926 | 0.932  (0.763, 1.118)  0.411 | 0.917  (0.769, 1.094)  0.336 | 1.196  (0.988, 1.448)  0.067 | 1.439^†^  (1.039, 1.993)  0.028 |
| Pain | RR  (95% CI)  p-value | 0.980  (0.926, 1.037)  0.481 | 0.906  (0.851, 0.964)  0.002 | 0.964  (0.924, 1.006)  0.088 | 0.9683  (0.906, 1.023)  0.218 | 0.931  (0.895, 0.968)  <0.001 | 1.018  (0.946, 1.095)  0.639 |
| Mobility | RR  (95% CI)  p-value | 1.001  (0.976, 1.043)  0.607 | 0.940  (0.907, 0.975)  0.01 | 0.992  (0.950, 1.036)  0.711 | 0.973  (0.940, 1.008)  0.130 | 0.948  (0.921, 0.975)  <0.001 | 1.047  (0.999, 1.096)  0.0.052 |
| Cognitive Impairment | $\beta^{b}$  (95% CI)  p-value | 0.106^†^  (0.003, 0.183)  0.011 | 0.126  (0.062, 0.190)  0.001 | 0.070^†^  (0.030, 0.110)  0.003 | 0.049^†^  (0.002, 0.096)  0.041 | 0.055^†^  (0.016, 0.093)  0.010 | 0.064^†^  (0.001, 0.127)  0.047 |
| All-cause mortality | OR  (95% CI)  p-value | 0.846^†^  (0.748, 0.957)  0.008 | 0.814  (0.744, 0.890)  <0.001 | 0.818^†^  (0.707, 0.945)  0.006 | 0.813  (0.732, 0.901)  <0.001 | 0.887  (0.769, 1.023)  0.099 | 0.864  (0.708, 1.056)  0.153 |

CI, confidence interval; OR, odds ratio; RR, risk ratio; ADL, activities of daily living; IADL, instrumental activities of daily living.

^a^ Missing covariate variables were imputed using chained equations (ten sets of imputed data were generated). All models were controlled for participant demographics: age, gender, marital status, educational attainment, and country; socioeconomic factors: annual personal income, household net financial assets. Each model was also adjusted for prior values of the 21 outcome variables and of the exposure variable, as well as previous self-reported presence/absence of diagnosis for heart attack, hypertension, high blood cholesterol, stroke, diabetes, and cancer simultaneously in each regression model.

^b^ All continuous outcomes were standardized (mean = 0, standard deviation = 1), and β was the standardized effect size.

^c^ p<0.05 after Bonferroni correction (p-value cut-off for Bonferroni correction = 0.05/21 outcomes=0.0024)

^†^ Not significant after Bonferroni correction.

**Table S5.** Robustness to Unmeasured Confounding (E-Values) for Assessing the Prospective Associations Between Reading Books, Magazines or Newspapers, Doing Word and Number Games and Playing Cards or Games and Well-Being, Physical Health, Daily Life Functioning, Cognitive Impairment and All-Cause Mortality (N =19,821). Survey of Health, Ageing and Retirement in Europe (SHARE), Adults Aged 50 and Over.

| **Outcome** | **Reading books, magazines and newspapers**  **(ref.= Never)** | | | | **Doing word or number games  (ref.= Never)** | | | | **Playing cards or games**  **(ref.= Never)** | | | |
| --- | --- | --- | --- | --- | --- | --- | --- | --- | --- | --- | --- | --- |
|  | **Sometimes^d^** | | **Almost every day** | | **Sometimes^d^** | | **Almost every day** | | **Sometimes^d^** | | **Almost every day** | |
|  | **E-Value  for Effect Estimate** | **E-Value for CI Limit** | **E-Value for Effect Estimate** | **E-Value for CI Limit** | **E-Value  for Effect Estimate** | **E-Value for CI Limit** | **E-Value for Effect Estimate** | **E-Value for CI Limit** | **E-Value  for Effect Estimate** | **E-Value for CI Limit** | **E-Value for Effect Estimate** | **E-Value for CI Limit** |
| Well-being |  |  |  |  |  |  |  |  |  |  |  |  |
| Loneliness (3-item loneliness scale) | na | na | 1.29 | 1.09 | na | na | na | na | 1.30 | 1.21 | 1.41 | 1.29 |
| Alzheimer’s disease | na | na | na | na | na | na | na | na | na | na | 2.69 | 1.52 |
| Depression (EURO-D≥4) | na | na | 1.38 | 1.08 | 1.39 | 1.15 | 1.37 | 1.11 | 1.45 | 1.21 | na | na |
| Future looks good | 1.32 | 1.20 | 1.41 | 1.29 | 1.26 | 1.09 | na | na | 1.25 | 1.12 | na | na |
| I feel full of energy these days | na | na | 1.38 | 1.22 | 1.24 | 1.12 | 1.28 | 1.14 | na | na | na | na |
| On balance, I look back on my life with a sense of happiness | na | na | 1.52 | 1.39 | na | na | na | na | na | na | 1.37 | 1.19 |
| I look forward to each day | 1.35 | 1.13 | 1.41 | 1.26 | 1.32 | 1.23 | na | na | 1.33 | 1.21 | na | na |
| I feel that my life has meaning | na | na | 1.44 | 1.31 | na | na | na | na | na | na | na | na |
| Daily life functioning |  |  |  |  |  |  |  |  |  |  |  |  |
| ADL  (at least 1 limitation) | na | na | 1.53 | 1.20 | na | na | na | na | na | na | na | na |
| IADL  (at least 1 limitation) | 1.43 | 1.12 | 1.42 | 1.23 | na | na | na | na | na | na | na | na |
| Physical health |  |  |  |  |  |  |  |  |  |  |  |  |
| Heart attack | na | na | na | na | na | na | na | na | na | na | na | na |
| Hypertension | na | na | na | na | na | na | na | na | na | na | na | na |
| High blood cholesterol | na | na | na | na | na | na | na | na | na | na | na | na |
| Stroke | na | na | na | na | na | na | na | na | na | na | na | na |
| Diabetes | na | na | na | na | na | na | na | na | na | na | na | na |
| Cancer | na | na | na | na | na | na | na | na | 1.72 | 1.13 | 2.27 | 1.36 |
| Pain | na | na | 1.30 | 1.07 | na | na | na | na | 1.30 | 1.15 | na | na |
| Mobility | na | na | na | na | na | na | na | na | 1.25 | 1.12 | na | na |
| Cognitive Impairment |  |  |  |  |  |  |  |  |  |  |  |  |
| Date Orientation | 1.35 | 1.12 | 1.35 | 1.16 | 1.27 | 1.13 | na | na | na | na | na | na |
| All-cause mortality | na | na | na | na | 1.58 | 1.03 | 1.57 | 1.27 | na | na | na | na |

CI, confidence interval; na, nonapplicable because the effect estimate was not significant; ADL, activities of daily living; IADL, instrumental activities of daily living; ‘Sometimes’ corresponds to almost every week, almost every month, or less often.

An unmeasured confounder would need to be associated with both date orientation and occasional (sometimes) reading by risk ratios of 1.35 each, above and beyond the measured covariates, to explain away fully the observed association between occasional (sometimes) reading and subsequent date orientation (at the 6-year follow-up). With respect to the E-value for the limit of the 95% CI, this unmeasured confounder would need to be associated with both occasional (sometimes) reading and subsequent date orientation by 1.12-fold each, above and beyond the measured covariates, to shift the lower limit of the CI for the observed association between occasional (sometimes) reading and subsequent date orientation to include the null value.

**References**

1. Börsch-Supan A, Brandt M, Hunkler C, Kneip T, Korbmacher J, Malter F, Schaan B, Stuck S, Zuber S. Data resource profile: The survey of health, ageing and retirement in europe (SHARE). *Int J Epidemiol* (2013) **42**:992–1001. doi:10.1093/ije/dyt088

2. Hughes ME, Waite LJ, Hawkley Louise C, Cacioppo JT. A Short Scale for Measuring Loneliness in Large Surveys: Results From Two Population-Based Studies. *Res Aging* (2004) **26**:655–672. doi:10.1109/ICIT.2017.7913066

3. Guerra M, Ferri C, Llibre J, Prina AM, Prince M. Psychometric properties of EURO-D, a geriatric depression scale: A cross-cultural validation study. *BMC Psychiatry* (2015) **15**:12. doi:10.1186/s12888-015-0390-4

4. Mehrbrodt T, Gruber S, Wagner M. *Scales and Multi-Item Indicators. SHARE Survey of Health, Ageing and Retirement in Europe*. (2019).

5. Prince MJ, Reischies F, Beekman ATF, Fuhrer R, Jonker C, Kivela SL, Lawlor BA, Lobo A, Magnusson H, Fichter M, et al. Development of the EURO-D scale - A European Union initiative to compare symptoms of depression in 14 European centres. *Br J Psychiatry* (1999) **174**:330–338. doi:10.1192/bjp.174.4.330

6. Chan KS, Kasper JD, Brandt J, Pezzin LE. Measurement equivalence in ADL and IADL difficulty across international surveys of aging: Findings from the HRS, SHARE, and ELSA. *Journals Gerontol - Ser B Psychol Sci Soc Sci* (2012) **67**:121–132. doi:10.1093/geronb/gbr133

7. Dewey ME, Prince MJ. “Cognitive Function,” in *Health, Ageing and Retirement in Europe. First Results from the Survey of Health, Ageing and Retirement in Europe*, eds. A. Börsch-Supan, A. Brugiavini, H. Jurges, J. P. Mackenbach, J. Siegrist, G. Weber doi:10.1007/978-981-287-080-3_243-1

8. Rammstedt B, John OP. Measuring personality in one minute or less: A 10-item short version of the Big Five Inventory in English and German. *J Res Pers* (2007) **41**:203–212. doi:10.1016/j.jrp.2006.02.001
